# Rotavirus: The Challenges Ahead

**DOI:** 10.4103/0970-0218.58382

**Published:** 2009-10

**Authors:** Paramita Sengupta

**Affiliations:** Department of Community Medicine, Christian Medical College, Ludhiana–141 008, Punjab, India

## Introduction

Diarrhea remains one of the most common illnesses of children worldwide.(1) Rotaviruses are the single most important etiologic agents of severe diarrhea among young children, responsible for 114 million cases, 25 - 55% of all hospital admissions for diarrhea and more than 610,000 deaths worldwide every year.(2) Rotavirus is ubiquitous and by the age of three to five years, 95% of the children worldwide would have been infected.(3) It is estimated that 82% of these deaths occur in developing countries (the third most common cause of death, with about two million deaths per year) equivalent to approximately 1,205 child deaths each day, because of poorer access to treatment and a greater prevalence of malnutrition.(2)

In India, 350,000 children, under five, die every year due to diarrheal diseases, out of which one-third is due to rotavirus gastroenteritis. The prevalence of rotavirus diarrhea in India has been found to vary between 5 and 71% in hospitalized children < 5 years of age, with acute gastroenteritis.(4) Apart from the primary burden of rotavirus disease on childrens' health (mortality and morbidity), rotavirus also has a significant economic impact, which can be both direct and indirect. Examples of direct costs include medical costs resulting from hospitalizations, out patient visits, personnel, facilities, diagnostics, and medication. Indirect costs include those incurred to society and households as a result of lost work time when parents are forced to stay home to take care of sick children – resulting in reduction of labor productivity and loss of wages. Intangible costs such as pain and suffering add to the burden.

A review of available preventive measures against rotavirus infection in relevance to India was hence undertaken.

### Rotavirus

The rotavirus genus belongs to Reoviridae family of viruses. Rotaviruses are highly contagious and the predominant mode of transmission is the fecal–oral route.(3) It remains stable and infective in human feces for up to one week and can survive for weeks in recreational or drinking water, hence nosocomial infections are also widespread. Person-to-person spread, via contaminated hands, is probably the most important means by which rotaviruses are disseminated in close communities, such as hospitals and homes. Transmission among non-toilet trained children in nurseries and day care centers is facilitated by direct close contact, as well as sharing contaminated food, drinks or toys.(5) Asymptomatic excretion of rotavirus occurs in half of the infected children before the onset of clinical symptoms, and persists in one-third of the children a week after the symptoms end. Rotavirus infection is preceded by an incubation period of 24 – 48 hours. Symptoms range from vomiting and mild watery diarrhea of short duration to severe gastroenteritis with life-threatening dehydration, secondary to gastrointestinal fluid loss, a problem associated with developing countries.

Significant rotavirus shedding occurs during an episode of diarrhea, but many infected individuals shed the virus without experiencing diarrhea. Respiratory transmission has been speculated because of (i) the high rates of infection in the first three years of life regardless of sanitary conditions, and (ii) the failure to document fecal oral transmission in several outbreaks of rotavirus over large geographic areas in the winter.(3,6) Published epidemiological studies covering 18 Indian cities (1996 – 2001) showed that G1 was the single most common serotype, and the burden of severe disease and hospitalization is borne predominantly by G1, G2, G3, G4, and G9.(7)

### Preventive Measures

Passive immunity from placentally transferred maternal antibodies and breast-feeding is thought to play a role in protection against rotaviral disease in young infants,(8) hence, breast-feeding is highly recommended. Rigorous infection-control practices, for example, a hand hygiene program in a US hospital more than halved the incidence of nosocomial infection in only three years.(9) However, rotavirus is relatively resistant to inactivation by commonly used clinical disinfectants and antiseptics. Plain soap for hand washing is ineffective against rotavirus, so use of waterless hand cleaning agent containing alcohol has been recommended.(5) Efforts to decrease the number of deaths due to diarrhea have targeted interventions to improve water quality and sanitation and introduce treatment programs based on oral rehydration therapy (ORT). ORT has been found to be effective, relatively simple to administer, and inexpensive at treating diarrhea, but shortfalls in its coverage and use limit the extent to which it can be expected to provide a solution to the problem of diarrhea, as it is often more difficult to successfully administer in severe vomiting,(10) a common manifestation of rotavirus disease. Although these efforts have decreased the mortality rate associated with infection due to bacterial and parasitic agents, they have been less effective in reducing rotavirus disease-associated morbidity and mortality.(11) A similar incidence of rotavirus gastroenteritis in both developed and developing countries suggests that the problem will not be controlled by these measures. Moreover, administration of antibiotics will not be of any help in case of rotaviral or any other viral diarrhea, leading to an increase of antibiotic-resistant strains.(12) Indeed, because of the magnitude of the global health burden attributed to rotavirus disease, the development of rotavirus vaccines is the first-line strategy for prevention.(11)

### Vaccines

The large burden of rotavirus disease has led many groups reviewing the need for new vaccines to select rotavirus, a high priority target for accelerated vaccine development. WHO's Diarrheal Disease Control Program was the first to make this recommendation, which has been reaffirmed repeatedly by other international review groups, such as the Children's Vaccine Initiative (1996), the Bill and Melinda Gates Foundation (1999), and the Global Alliance for Vaccines and Immunization (GAVI).(13) In 1999, a highly efficacious rotavirus vaccine, RotaShield™ licensed in the United States, was withdrawn from the market after less than one year because of its association with intussusception.(14) There are two new live, oral, attenuated rotavirus vaccines: the monovalent human rotavirus vaccine (Rotarix™) produced by Glaxo Smith Kline Biologicals and the pentavalent bovine–human, reassortant vaccine (RotaTeq™) developed by Merck and Co. Both vaccines have demonstrated very good safety and efficacy profiles in large clinical trials in western industrialized countries and in Latin America.(3) Careful surveillance has not revealed any increased risk of intussusception in the vaccinated groups with either vaccine.(15) The new rotavirus vaccines are now introduced for routine use in a number of industrialized and developing countries. In general, they provide about 90 – 100% protection against severe rotavirus disease and about 74 – 85% protection against rotavirus diarrhea of any severity, depending on the schedule of administration and the population evaluated. The protection against severe rotavirus infection is shown to extend into the second year of follow-up for both these vaccines.(15)

The rationale for vaccination is based on the fact that after the first rotaviral infection, 88% of the children are protected against severe gastroenteritis;(16) which not only induces an immune response to the specific serotype involved, but also to the additional serotypes (heterotypic immunity).(16) Following the second infection, virtually all children are protected against severe disease.(16) Hence, the goal of rotavirus vaccination is to mimic or even exceed the immune response to natural infection, thus preventing severe diarrhea and its sequel (e.g., dehydration, physician visits, and hospitalizations).

Rotarix™ demonstrated an 86 – 90% efficacy against severe cases of Rotavirus gastroenteritis of any severity, RotaTeq™ demonstrated 75% efficacy against any Rotavirus gastroenteritis and 100% against severe forms.(15) Rotarix™ was licensed for sale by the Mexican authorities in July 2004, making it the first country in the world to introduce a new Rotavirus vaccine.(17) Rotavirus vaccines have the potential to benefit affected families by reducing lost wages and out-of-pocket costs of obtaining treatment. The US FDA had approved Rotarix in February 2008, for marketing.

The Rotarix™ vaccine is administered orally in a two-dose schedule to infants approximately two and four months of age. The first dose can be administered at the age of six weeks and must be given no later than the age of 12 weeks. The interval between the two doses should be at least four weeks. The two-dose schedule should be completed by age 16 weeks and not later than 24 weeks of age. For RotaTeq™, the recommended schedule is three oral doses at ages two, four, and six months. The first dose should be administered between ages 6 – 12 weeks and subsequent doses at intervals of 4 – 10 weeks. Vaccination should not be initiated for infants aged > 12 weeks. All three doses should be administered before the age of 32 weeks. There is a potentially higher risk of intussusception when the first dose of these vaccines is given to infants aged > 12 weeks; consequently, current rotavirus vaccines should not be used in catch-up vaccination campaigns, where the exact age of the vaccinees may be difficult to ascertain.(15) Simultaneous administration of rotavirus vaccines and other vaccines of the infant immunization program, including oral poliovirus vaccine, has not been seen to interfere significantly with the protective immune responses, following complete courses of the respective vaccines. Also, breast-feeding and prematurity (< 37 weeks' gestation) do not significantly impair the immune response to the rotavirus vaccines.

## Conclusion

The two main components of an economic evaluation of a rotavirus vaccine include: (i) the inputs or resources consumed as a result of rotavirus disease, (direct, indirect, intangible costs); and (ii) the outcomes, improvements in health (deaths prevented, hospitalizations prevented) resulting from the healthcare program (rotavirus vaccination). Despite marked improvements in water quality, sanitation, and hygiene, the fact that the incidence of rotavirus diarrhea has not decreased substantially during the past decade, suggests that immunization may be the most promising preventive strategy.(13) Preliminary data from the first three years of surveillance in Asia have already documented that in most countries of the region, more than 40 –50% of all children admitted for diarrhea have rotavirus as their pathogen.(18)

By the time the Asian birth cohort reaches five years of age, it is estimated that 171,000 children will die, 1.9 million will be hospitalized, and 13.5 million will require outpatient care from rotavirus diarrhea, for an associated healthcare cost of more than US $ 191 million. A universal rotavirus immunization program has the potential to avert 109,000 deaths, 1.4 million hospitalizations, 7.7 million outpatient visits, and US $ 139 million in healthcare costs each year, for children < 5 years of age, in Asia.(19)

In a study on 63,225 healthy infants from 11 Latin American countries and Finland, the efficacy of Rotarix vaccine against severe rotavirus gastroenteritis and against rotavirus hospitalization was 85% (*P* < 0.001 for the comparison with placebo) and reached 100% against more severe rotavirus gastroenteritis. Hospitalization for diarrhea of any cause was reduced by 42% (*P* < 0.001).(20)

Rotavirus vaccines will provide protection only in case of childhood diarrhea. Therefore, as Glass and colleagues state, *“there is a nagging question as to whether mothers might feel cheated or misled if their child received a RV vaccine, but still developed diarrhea, albeit of a different etiology.”*(11)

However, even if economic analyses suggest that RV vaccines represent good value for money, an additional challenge will be to ensure that the funding mechanisms are in place.

An acceptable price is established according to the value of the vaccine need, including the disease burden, impact of the vaccine, and cost-effectiveness relative to other interventions. Once an acceptable price has been established, the question of affordability ensues. The cost of the Rotarix™ vaccine in India is Rs. 999/-. It has great potential for saving lives but its exorbitant price (Rs. 2000/- for two doses needed to protect a child) drives it out of reach of the common man. International effort (which is done for polio vaccine) and domestic manufacture may help a lot in developing countries, where the disease burden is enormous. The Vaccine Fund and the GAVI have committed to purchase vaccines for 74 poorest countries of the world.(11)

A key aim of the Millennium Development Goals was a reduction of mortality, by two-thirds, in children younger than five years from the level, in 1990.(21) Inclusion of rotavirus vaccines into the routine schedule for childhood immunization would be an important addition to prevent 5% of all childhood deaths caused by rotavirus, and as much as 40% of all deaths due to diarrheal diseases. Enhanced efforts to bring these vaccines to all children could go a long way in achieving these important and common goals. In developing countries, introduction of these vaccines could, in addition, reduce the heavy burden of severe rotavirus diarrhea and prevent a large proportion of the estimated 527 000 annual deaths caused by such disease in young children, and thereby contribute to reducing the global under-five mortality (Goal 4 of the Millennium Development Goals). Although an intervention may not be cost saving, it may be considered cost-effective if the health benefits of the intervention outweigh the financial investment required to implement the intervention.(19)
